# Epidemiology of Pediatric Tuberculosis and Factors Associated with Unsuccessful Treatment Outcomes in the Centre Region of Cameroon: A Three-Year Retrospective Cohort Study

**DOI:** 10.1155/2022/2236110

**Published:** 2022-08-24

**Authors:** Thomas Achombwom Vukugah, Derick Akompab Akoku, Micheline Mekemnang Tchoupa, Edward Lambert

**Affiliations:** ^1^Atlantic International University, Honolulu, HI, USA; ^2^Health Alliance International, Abidjan, Côte d'Ivoire; ^3^Department of Global Health, University of Washington, Seattle, WA, USA; ^4^Ministry of Public Health, Yaoundé, Cameroon

## Abstract

**Background:**

In Cameroon, there are limited data on treatment outcomes of pediatric tuberculosis (TB). We sought to identify the factors associated with unsuccessful treatment outcomes and the risk factors for mortality among children receiving TB treatment in the Centre Region of Cameroon.

**Methods:**

This was a multicentre facility-based retrospective cohort study using routinely collected programmatic data. All children <15 years old treated for TB between 2018 and 2020 in 21 health facilities were included. We assessed risk factors for experiencing an unsuccessful treatment outcome and mortality through multivariable logistic regression analysis.

**Results:**

Of the 610 children with TB, 307 (50.3%) were females and the median age was 6 years (IQR = 2–12). One hundred and fifty-three (25.1%) of the children were TB/HIV co-infected patients. TB treatment success (cases categorized as cured and completed treatment) was observed in 488 (80.0%) of the patients. Unsuccessful treatment outcomes were experienced by 122 (20.0%) children. Of these, 73 (12.0%) died, 4 (0.6%) had treatment failure, 25 (4.1%) were lost to follow-up, and the outcomes of 20 (3.3%) children were not evaluated. In multivariable analysis, HIV-positive status (adjusted odds ratio [AOR] = 2.43; 95% CI, 1.55–3.80, *p* < 0.001) and clinical method of TB diagnosis (AOR = 2.46; 95% CI, 1.55–3.91, *p* < 0.001] were associated with unsuccessful treatment outcomes. HIV-positive status (AOR = 4.23; 95% CI, 2.44–7.33, *p* < 0.001) and clinical method of TB diagnosis (AOR = 2.22; 95% CI, 1.25–3.91, *p*=0.006) were the risk factors for mortality among children on TB treatment.

**Conclusion:**

The study found that HIV-TB co-infected children and those clinically diagnosed with TB were significantly more likely to have had unsuccessful TB treatment outcomes and mortality. Our findings underscore the need for healthcare workers to closely monitor and support HIV-TB co-infected children on TB treatment. TB/HIV collaborative activities should be strengthened by implementing TB preventive interventions among HIV-infected children.

## 1. Introduction

Globally, millions of children are exposed to *Mycobacterium tuberculosis* (TB) each year, and TB remains a leading cause of morbidity and mortality in children worldwide [1, 2]. The World Health Organization (WHO) estimates that 1.12 million children globally develop TB annually, with approximately 200,000 TB deaths despite the availability of effective TB prevention, treatment, and universal vaccination at birth in high-TB-burden countries [3, 4]. The diagnosis of TB in children is challenging. The paucibacillary nature of TB in children contributes to poor sensitivity of microbiological tests; difficulties in obtaining respiratory samples, especially since young children under the age of 5 are unable to self-expectorate; poor specificity of symptoms, particularly in those with HIV infection or severe acute malnutrition; and complicated chest radiography (CXR) features that are difficult to diagnose [5–7]. Young, malnourished, or human immune deficiency virus (HIV)-infected children exposed to *Mycobacterium* TB are at disproportionately high risk of developing disseminated TB or dying [8,9].

Cameroon is among the 30 highest TB and TB/HIV burden countries with an estimated 22,499 cases of all forms of TB detected across the country in 2020 [10]. Of these, 1,158 (5.2%) were diagnosed among children under 15 years of age, which suggest that 50% of pediatric TB cases are not diagnosed [10]. TB in the country is worsened by the HIV epidemic as it is the most common opportunistic infection among people living with HIV (PLHIV). In Cameroon, the estimated prevalence of HIV in the general population is 2.7% [11]. In 2020, about 6,074 (27%) of the total number of TB cases were co-infected with HIV [10].

Surveillance data on pediatric TB are vital for understanding its epidemiology and gaining insights into the factors associated with unsuccessful TB treatment outcomes. A variety of clinical, social, and economic factors have been identified as important factors associated with TB treatment outcomes in children in high-TB countries [12]. The most commonly reported factors associated with unsuccessful treatment outcomes for pediatric TB include adherence to anti-TB treatment, low body weight, HIV-positive status, male sex, clinical and/or bacteriological method of diagnosis, pulmonary positive case, unknown HIV status, type of health facility, pretreatment sputum smear, and household contact with cases of TB treatment failure [12–23].

Despite Cameroon's commitment to TB eradication, pediatric TB diagnosis and management remain difficult. In the centre region of the country, 140 (12.1%) pediatric TB cases were diagnosed in 2020 [10]. Since 2016, the National Tuberculosis Control Program (NTCP) at the Ministry of Health (MOH) has invested resources in the early introduction and application of the new dispersible first-line fixed-dose combination (FDC) therapy for the treatment of children. While studies on childhood treatment outcomes have been conducted in the other sub-Saharan African countries [13, 15, 20, 24, 25], there is a limited published study that has evaluated TB treatment outcomes among children in Cameroon. The absence of such data makes it difficult for health professionals and the NTCP to determine the effectiveness of TB treatment among children and identify factors that contribute to unsuccessful treatment outcomes to guide the formulation of policies and interventions to address this public health problem. The objectives of this study were (1) to identify the factors associated with unsuccessful treatment outcomes among children on TB treatment and (2) to investigate the risk factors for mortality among children on TB treatment in the Centre Region of Cameroon. The findings of this study will inform policy, planning, and programmatic action, including the design and implementation of targeted interventions for this vulnerable population.

## 2. Methods

### 2.1. Study Design and Population

This study was a multicentre facility-based retrospective cohort study using routinely collected programmatic data. All children (<15 years of age) diagnosed with and treated for TB between January 1, 2018, to December 31, 2020, in each of the selected health facilities, and registered were considered in the study. Children aged 15 years and above, with ongoing treatment or with incomplete data on treatment outcomes, were excluded.

### 2.2. Sampling of the Health Facilities

The sampling frame was the list of the 63 health facilities known as Diagnostic and Treatment Centres (DTCs) in the region. We applied a modified version of the multistage sampling technique to select health facilities that participated in the study. In the 1st stage, all the 63 DTCs in the region were stratified into three categories (public, private, and faith-based). Stratification was performed to increase precision by grouping categories within the sample into more homogeneous groups. At the end of stratification, there were 38 health facilities in the public category; seven in the private category, and 18 in the faith-based category.

Based on time and available resources, the study team agreed that data should be collected from one-third (*n* = 21) of the 63 health facilities providing TB care and treatment in the region. Consequently, in the 2nd stage, simple random sampling was used to select health facilities from each of the three categories based on probability proportional to size. At the end of the random selection process, 13 health facilities, were selected from public sector facilities, six from faith-based facilities, and two from private health facilities, giving a total of 21 health facilities.

### 2.3. Sample Size Determination

The sample size was estimated using the single population proportion formula for cross-sectional studies. The minimum sample size was derived from the formula: *n* = [*z*^2^ × *p* (1 − *p*) DEFF]/[m^2^], where *n* is the minimum required sample size for the study; *z* is the standard deviation for a 2-tailed test at a 95% confidence level (1.96); *p* (50%) is an estimate of the unsuccessful treatment rate (as this expected proportion *p* produces the largest sample size (for a given value of *m* and DEFF); *m* is the margin of error (5%), and DEFF = 1.5 is the estimated design effect, which gives a sample size of 576 participants. Note *p*=50% is the assumed unsuccessful treatment rate for pediatric TB cases given the nonavailability of such data in the country at the time of the study. After accounting for a 5% nonresponse rate, the minimum estimated sample size was 605, which was required to build sound statistical conclusions and inferences.

### 2.4. Measurement

#### 2.4.1. Dependent Variables

Based on the objectives of this study, there were two outcome variables: TB treatment outcome and child mortality.

TB treatment outcome was a binary variable of either successful or unsuccessful treatment outcome. All children who completed their TB treatment and those cured were considered “successful treatment outcome.” Those who defaulted the treatment (not evaluated), were lost to follow-up (LTFU), died, and had a recorded treatment failure were classified as “unsuccessfully treatment outcome.” Treatment outcome categories (cured and treatment completed, died, treatment failure, LTFU, and not evaluated) were defined based on the NTCP guidelines by the current WHO guidelines.

Child mortality was a binary variable with either dead or alive outcomes. Any child who died at any moment between diagnosis and foreseen the end of treatment for any reason was considered “died,” while those living during or at the end of treatment irrespective of outcome were considered “alive.”

#### 2.4.2. Independent Variables

The independent variables included age of the child (0–4/5–9/10–14), sex (male/female), treatment protocol, residence (urban/rural), HIV status (positive, negative, unknown), year of TB diagnosis, clinical features of TB (smear-positive pulmonary TB/smear-negative pulmonary TB/extra pulmonary TB), body weight at TB treatment initiation, method of TB diagnosis (bacteriological and clinical), and type of health facility (public, private, or faith-based). TB diagnosis was based on standard WHO/NTCP methods [26] using any sputum microscopy, clinical examination, chest X-ray, TB-Lamp, and GeneXpert for diagnosis.

### 2.5. Operational Definitions

Table 1 shows the definition of the TB treatment outcomes used in this study, which was based on the standard operational definitions of the NTCP.

### 2.6. Data Collection Procedures

Data collection took place from January to February 2022 by data clerks who were trained on how to extract data from TB records in the 21 selected health facilities. A paper-based data extraction tool was developed, which mirrored the National TB Treatment register and TB treatment card that was used to extract data. Data on all pediatric TB cases registered from January 2018 to December 2020 were extracted from the TB treatment cards and TB Treatment registers into a data extraction tool by trained data clerks. They extracted sociodemographic and clinical data of children treated for TB who met the eligibility criteria in each selected health facility. All data in the extraction tool were later entered into mobile tablet devices using Open Data Kit (ODK) software. The study team strictly monitored data collection daily to ensure the completeness of the abstracted data. At the end of each day, the data clerks synced all data in their mobile tablets to a Google Drive Server that was developed.

### 2.7. Statistical Analysis

The dataset was downloaded from the Google Server in an excel format, verified for completeness and consistency, and later exported into the Statistical Package for Social Science (SPSS) software version 23.0 (IBM, Armonk, NY, USA) for analysis. Patients with incomplete data on treatment outcomes were excluded from the analysis. For the TB treatment outcome variable, unsuccessful treatment was coded as “1,” while successful treatment was coded as “0.” For the child mortality outcome variable, “died” was coded as “1,” while “alive” was coded as “0.” Descriptive statistics were used to determine the characteristics of the study population. Demographic and clinical characteristics and treatment outcome variables were compared in bivariate analysis using the chi-squared (*χ*^2^) test. Variables that were significantly associated with unsuccessful treatment at *p*-value <0.25 in bivariate analysis were considered candidates for logistic regression analyses. Univariate and backward stepwise multivariate logistic regression analyses were performed to estimate the strength of the associations between the outcome and the variables that were significant in univariate analyses.

Risk factors for child mortality were also assessed in univariate and multivariate analysis. Children (aged <15 years) who died during treatment were compared to those who were alive at the end of treatment regardless of whether they were cured, or completed their treatment, or had failed treatment. We did not consider children who were LTFU in this definition. Given that the cause-of-death data are not accurately reported in the Cameroon TB surveillance system, we analyzed all deaths reported among children who were receiving TB treatment, rather than only deaths reported as caused by TB. We performed univariate logistic regression to identify risk factors associated with death. All variables that were significant in the univariate analysis were entered in the multivariate-adjusted model. Adjusted odds ratio (AOR) with their 95% confidence intervals was estimated, and the level of statistical significance was set at *p* < 0.05.

## 3. Results

### 3.1. Sociodemographic and Clinical Characteristics of the Study Participants

Overall, 622 children were diagnosed with TB in the 21 health facilities during the study period. Of these, 12 children were excluded due to missing data, and 610 children were considered for analysis.  Table 2 shows participants' demographic and clinical characteristics by treatment outcome. The median age of the children was 6 years (IQR = 2–12), and 307 (50.3%) were females. Most, 463 (75.9%) of the children lived in urban areas, while the rest lived in rural areas. Two hundred and forty-five (40.2%) of the children were diagnosed positive for pulmonary TB. Three hundred and sixty-five (59.8%) of the children were clinically diagnosed, while 132 (21.6%) weighed between 8 to 14 kg at the time of TB initiation. One fifty-three (25.1%) of the children were TB/HIV co-infected patients. Four hundred and twenty-two (69.2%) of the children received the pediatric treatment regimen (2RHEZ+4RH), while 188 (30.8%) received the adult regimen (2RHEZ + 4RH). Three hundred and eleven (51.0%) of the children started TB treatment in 2020, while 146 (23.9%) started treatment in 2019.

The overall treatment success rate was 488 (80.0%), while 122 (20.0%) had unsuccessful treatment outcomes. Of the 610 children enrolled, patients who were clinically diagnosed and HIV-positive had higher unsuccessful treatment outcomes than bacteriologically diagnosed (*p* < 0.001) and HIV-negative patients (*p* < 0.001), respectively. Children who were on the adult regimen had higher unsuccessful treatment outcomes than those on the pediatric regimen (*p*=0.02). Children who had extrapulmonary TB (EPTB) had higher unsuccessful outcomes than those who had pulmonary TB (*p* < 0.001). Unsuccessful treatment outcomes among female patients were higher compared with male patients, but this difference was not statistically significant. There were no significant differences in unsuccessful treatment outcomes between the age of the child (*p*=0.72) and the weight of the child at TB initiation (*p*=0.75).

## 4. Treatment Outcome of Pediatric TB Patients

Table 3 shows the distribution of treatment outcomes and TB types in children in the three years (2018–2020). Overall, 488 (80.0%) of the children had successful treatment outcomes; 351 (57.5%) of whom completed treatment, and 137 (22.5%) were cured. The trend of the treatment outcome revealed an increment in the treatment success rate during the study period ranging from 115 (77.2%) in 2018 to 258 (80.0%) in 2020.

The overall unsuccessful treatment rate was 122 (20.0%). Of these, 73 (12.0%) died. Very few, 4 (0.6%), children had treatment failure. LTFU cases were observed in 25 (4.1%) of children and not-evaluated children were at 20 (3.3%). The trend of EPTB cases increased steadily across the study period from 36 (23.5%) in 2018 to 100 (32.2%) in 2020. The trend of clinically diagnosed pulmonary TB (PTB−) cases varied across the study period from 49 (32.0%) in 2018 to 97 (31.2%) in 2020. This was the same with bacteriologically confirmed pulmonary TB (PTB+) cases, 68 (44.4%) in 2018 to 114 (36.7%) in 2020.

### 4.1. Factors Associated with Unsuccessful Treatment Outcome

Table 4 shows the association of demographic and clinical characteristics with unsuccessful treatment outcomes. In the univariate model, type of TB, method of diagnosis, HIV status, treatment protocol, and year of TB initiation were significantly associated with unsuccessful treatment outcomes.

In the multivariate model, children with the clinical method of TB diagnosis were significantly more likely (AOR = 2.46; 95% CI, 1.55–3.91, *p* < 0.001] to have had unsuccessful treatment outcomes compared to those who were bacteriologically diagnosed. HIV-positive children were significantly more likely (AOR = 2.43; 95% CI, 1.55–3.80, *p* < 0.001) to have had unsuccessful treatment outcomes compared to those who were HIV-negative.

### 4.2. Factors Associated with Mortality among Pediatric TB Cases

Table 5 shows the association of demographic and clinical characteristics with mortality among pediatric TB cases. Of the 610 children in the analysis, 73 (12.0%) died during treatment, while 512 (83.9%) were alive. In the univariate model, the form of TB, method of diagnosis, the weight of the child at TB initiation, and HIV status were significantly associated with mortality. After adjusting for confounders, children with the clinical method of TB diagnosis were significantly more likely (AOR = 2.22; 95% CI, 1.25–3.91, *p*=0.006) to have died compared to children bacteriologically diagnosed. HIV-positive children were significantly more likely (AOR = 4.23; 95% CI, 2.44–7.33, *p* < 0.001] to have died compared to HIV-negative children.

## 5. Discussion

The purpose of this study was to identify the factors associated with unsuccessful treatment outcomes and the risk factors for mortality among children receiving TB treatment in the Centre Region of Cameroon. This study was relevant because it is among the very few studies that have investigated TB treatment outcomes among children in the country.

In this current study, 254 (41.6%) of the children were between the ages of 0 and 4 years. This allays concerns about potential under-notification in the 0–4-year age group and is supported by the expected epidemiology of pediatric TB. This is in contrast to other studies from Nigeria [13], Ethiopia [15], and Pakistan [19], which found very few cases of pediatric TB in children aged 0 to 4 years. This may be because of the under-reporting of TB in children aged 0–4 years as a result of diagnostic challenges. The majority, 245 (40.2%) of the children were diagnosed with PTB+, and the minority, 177 (29%) had EPTB, which is a usual distribution. The authors from other limited resource countries have reported similar results [14, 19]. Other authors, however, reported higher rates of EPTB and PTB- in Nigeria [13] and Ethiopia [20], which can be attributed to variations in clinical presentation and difficulties with case management.

Our study found that 122 (20.0%) of children on TB treatment during the study period had unsuccessful treatment outcomes. This proportion is considered to be high based on the national average and the WHO target of a 10% unsuccessful treatment rate [26, 27]. Our findings are in contrast to studies conducted in Malaysia, Ethiopia, Nigeria, and Uganda, which reported 9.9% to 18.0% unsuccessful treatment outcomes [13, 14, 20, 28]. However, a previous study conducted in the Democratic Republic of Congo found a high 86 (30.4%) proportion of unsuccessful treatment outcomes [24]. The high unsuccessful treatment outcomes in this study could be explained by the high number of children who died, those who were LTFU, who were not evaluated, and who had failed treatment, which was high in this study 49 (40.1%) compared to other studies [14, 15, 17]. This finding suggests that patient follow-up or treatment supportive supervision has been suboptimal in the centre region. Furthermore, these results could be attributed to inadequate implementation of the treatment guidelines by TB frontline healthcare workers (HCWs).

In this study, children who were clinically diagnosed were more likely to have had unsuccessful treatment outcomes and a previous study conducted in Nigeria reported similar findings [13]. This finding is contrary to previous studies in Myanmar and Pakistan, which suggested that the bacteriological method of diagnosis was associated with unsuccessful treatment outcomes [16, 19]. It was not possible to obtain data on the exact type of clinical diagnostic test that the children underwent before being diagnosed with TB. However, one study in Malaysia identified that advanced chest X-ray findings were associated with successful treatment outcomes [29].

We also found that children with HIV-positive status were more likely to have had unsuccessful treatment outcomes with similar findings previously reported in Nigeria, Côte d'Ivoire, Zambia, and Peru [13, 30–32]. A possible explanation could be due to the predominance of respiratory comorbidity, which increases the risk of delayed diagnosis and treatment, missed diagnosis, atypical presentation, and mortality in HIV co-infected and disseminated TB children, particularly those with advanced HIV disease [33, 34]. The HIV co-infected children would require closer monitoring in an effective program.

We found that 73 (12.0%) of children on treatment died within the study period, a proportion which is considered to be high compared to the national target, which is ≤5% of the total TB cases [26]. This finding is in contrast to studies conducted in Kenya, Ethiopia, and South Africa, which found that less than 5% of children died during TB treatment [15, 17, 35]. Studies conducted in Mozambique, Tanzania, and Malawi reported high death outcomes in children with TB of 10%, 10.9%, and 17%, respectively [12, 36, 37]. The high proportion of death found in this study could be explained by the delay in pediatric TB diagnosis due to the presentation of non-specific symptoms in children, especially among HIV/TB co-infected, malnourished, and pneumonia children making bacteriological diagnosis very difficult. Waiting for a clinical diagnosis may have delayed treatment initiation, and by the time children clinically diagnosed with TB disease have reached a severe stage [13, 19, 38]. Furthermore, given that the cause-of-death data are not accurately reported in the Cameroon TB surveillance system, the proportion of children who died might be high due to reasons other than TB.

This study found that the HIV status and method of diagnosis were predictors of death. HIV-positive children were significantly more likely to have died compared to HIV-negative children. HIV-infected children experience faster disease progression and are predisposed to poor treatment response [33, 39, 40]. In this study, children with the clinical method of TB diagnosis were significantly more likely to have died compared to children bacteriologically diagnosed. A substantial proportion of children treated for TB were not subjected to any diagnostic testing. This finding may be attributed to the difficulty in obtaining sputum specimens for testing in children and the limited availability of radiography services. Consequently, these children were diagnosed clinically. A clinical diagnosis of TB relies on scoring systems and the acumen of clinicians, which is not standardized [17, 41]. This is a potential cause of bias, because children on treatment for clinically diagnosed TB (especially severest cases of TB) may have had other diseases that could drive mortality. Improving the coverage of HIV interventions for children and the coordination between TB and HIV services is essential to reducing TB-associated mortality in children. In addition, we observed that the method of diagnosis and the HIV status were common among the two study outcomes (unsuccessful treatment and mortality). A possible explanation is that HIV infection is a known risk factor for mortality among TB patients and also for unsuccessful treatment outcomes so not surprised with the results. With regard to the method of diagnosis, it is an opportunity for further research.

The findings of this study should be interpreted taking into consideration some limitations. Our study relies on routinely collected programmatic data, and variables such as treatment adherence and other disease conditions, as well as economic, behavioral, and social factors, which might have effect treatment outcomes were not routinely captured. Secondly, the causal relationship between the dependent and independent variables cannot be established because this was an observational study. Lastly, the findings of this study cannot be generalized to the entire pediatric population in Cameroon. Nevertheless, our study highlights the effectiveness of pediatric TB management and identified gaps in the TB program, which helps TB partners and MOH to design and implement targeted interventions for this vulnerable population. Future studies could be conducted at the national level, and survival analysis should be performed to estimate the cumulative incidence of death if data on the date of death and data of LTFU are known.

## 6. Conclusion

This study found an overall unsuccessful TB treatment rate of 122 (20.0%), with 73 (12.0%) of the pediatric cohort dying. TB/HIV co-infection is high among children on treatment for TB. The study also showed that HIV positivity and clinical method of diagnosis were associated with unsuccessful treatment outcomes and mortality in children. These findings provide relevant information to NTCP and MOH and underscore the need to enhance effective supportive supervision across health facilities. In addition, proper follow-up of children on TB treatment by HCWs at both health facility and community levels with an ultimate goal of optimizing successful treatment outcomes is required. Routine screening for TB and comorbidities such as HIV should also be intensified among children aged 0–14 years. HIV-co-infected children would require closer monitoring in an effective program and HIV/TB care should remain a national/international priority.

## Figures and Tables

**Table 1:** Treatment outcomes and operational definitions.

Type of TB treatment outcome	Definition	Classification/study outcome
Cured	A child diagnosed at the beginning of treatment as PTB + who is smear-negative in the last month of treatment and on at least one previous occasion at least 30 days apart	Successful treatment outcome
Completed treatment	A child who has completed treatment but does not have a negative smear in the last month of treatment and on at least one previous occasion more than 30 days prior. The smear examination may not have been done, or the results may not be available at the end of treatment.	
Treatment failure	A patient whose baseline smear at the beginning of treatment was positive and remains or becomes positive again at 5 months or later during treatment. This definition excludes those patients who are diagnosed with rifampicin or multidrug-resistant tuberculosis.	Unsuccessful treatment outcome
Not evaluated	A child on TB treatment for whom no treatment outcome is assigned. This includes cases “transferred out” to another treatment unit as well as those whose treatment outcome is unknown at the health facility.	
Lost to follow-up (LTFU)	When a child took TB treatment for at least 1 full month, treatment was interrupted for two consecutive months or more during the treatment period.	
Died	A child who dies at any moment between diagnosis and successful discharge from the treatment program.	

**Table 2:** Demographic and clinical characteristics by treatment outcome.

Demographic and clinical characteristics	Total sample *N* (%)	Unsuccessful outcome *N* (%)	Successful outcome *N* (%)	*p*-value^1^
Sample size	610 (100)	122 (20)	488 (80)	

Gender				
Female	307 (50.3)	64 (52.5)	243 (49.8)	0.599
Male	303 (49.7)	58 (47.5)	245 (50.2)	

Age group (years)				
0–4	254 (41.6)	53 (43.4)	201 (41.2)	0.721
5–9	142 (23.3)	30 (24.6)	112 (23.0)	
10–14	214 (35.1)	39 (32.0)	175 (35.9)	

Residence				
Rural	147 (24.1)	22 (18.0)	125 (25.6)	0.080
Urban	463 (75.9)	100 (82.0)	363 (74.4)	

Form of TB				
Extrapulmonary TB	177 (29.0)	39 (32.0)	138 (28.3)	<0.001
PTB−	188 (30.8)	53 (43.4)	135 (27.7)	
PTB+	245 (40.2)	29 (24.6)	216 (44.1)	

Method of diagnosis				
Bacteriological diagnosis	245 (40.2)	31 (25.4)	214 (43.9)	<0.001
Clinical diagnosis	365 (59.8)	91 (74.6)	274 (56.1)	

Weight of child at TB initiation				
0–7 kg	84 (13.8)	21 (17.2)	63 (12.9)	0.757
8–14 kg	132 (21.6)	28 (23.0)	104 (21.3)	
15–21 kg	124 (20.3)	22 (18.0)	102 (20.9)	
22–28 kg	73 (12.0)	16 (13.1)	57 (11.7)	
29–35 kg	88 (14.4)	16 (13.1)	72 (14.8)	
>35 kg	109 (17.9)	19 (15.6)	90 (18.4)	

HIV status				
HIV-negative	310 (50.8)	59 (41.4)	251 (51.4)	<0.001
HIV-positive	153 (25.1)	53 (43.4)	100 (20.5)	
Unknown	147 (24.1)	10 (8.2)	137 (28.1)	

Treatment protocol				
Pediatric regimen (2RHEZ +4RH)	422 (69.2)	74 (60.7)	348 (71.3)	0.023
Adult regimen (2RHEZ +4RH)	188 (30.8)	48 (39.3)	140 (28.7)	

Year of TB initiation				
2018	153 (25.1)	38 (31.1)	115 (23.6)	0.130
2019	146 (23.9)	31 (25.4)	115 (23.6)	
2020	311 (51.0)	53 (43.4)	258 (52.9)	

Type of health facility				
Faith-based	49 (8.0)	6 (4.9)	43 (8.8)	0.302
Private	19 (3.1)	5 (4.1)	14 (2.9)	
Public	542 (88.9)	111 (91.0)	431 (88.3)	

^1^ *p*-value from *X*^2^ test. PTB+: bacteriologically confirmed pulmonary TB. PTB−: clinically diagnosed pulmonary TB.

**Table 3:** Distribution of treatment outcomes and TB types in children in the three years (2018–2020).

Treatment outcome and type of TB	Time in years			Total
	2018 *N* (%)	2019 *N* (%)	2020 *N* (%)	
Successful	115 (77.2)	115 (78.8)	258 (80)	488 (80)
Completed	74 (48.4)	74 (50.7)	203 (57.5)	351 (57.5)
Cured	41 (28.8)	41 (28.1)	55 (22.5)	137 (22.5)

Unsuccessful	38 (24.8)	31 (21.2)	53 (17.6)	122 (20)
Died	22 (14.4)	17 (11.6)	34 (12.0)	73 (12.0)
Failure	0 (0.0)	0 (0.0)	4 (0.7)	4 (0.6)
Lost to followup	8 (5.2)	5 (3.4)	12 (3.9)	25 (4.1)
Not evaluated	8 (5.2)	9 (6.2)	3 (1.0)	20 (3.3)

Total type of TB	153	146	311	610
EPTB	36 (23.5)	41 (28.1)	100 (32.2)	177 (29.0)
PTB−	49 (32.0)	42 (28.8)	97 (31.2)	188 (30.8)
PTB+	68 (44.4)	63 (43.2)	114 (36.7)	245 (40.2)

EPTB extra pulmonary TB, PTB+ bacteriologically confirmed pulmonary TB, PTB− clinically diagnosed pulmonary TB.

**Table 4:** Factors associated with unsuccessful treatment outcome.

Demographic and clinical factors	Univariate analysis		Multivariate analysis	
	COR (95% CI)	*p*-value	AOR (95% CI)	*p*-value
Residence				
Rural	0.63 (0.38–1.05)	0.082	0.70 (0.41–1.22)	0.208
Urban	1	1

Type of TB				
Extra pulmonary TB	2.02 (1.20–3.41)	0.008	1.31 (0.41–4.24)	0.588
PTB−	2.81 (1.71–4.62)	<0.001	1.41 (0.44–4.47)	0.554
PTB+	1	1

Method of diagnosis				
Clinical diagnosis	2.29 (1.46–3.57)	<0.001	2.46 (1.55–3.91)	<0.001
Bacteriological diagnosis	1	1

HIV status				
HIV-positive	2.25 (1.45–3.49)	<0.001	2.43 (1.55–3.80)	<0.001
Unknown	0.31 (0.15–0.62)	0.001	0.32 (0.16–0.64)	0.001
HIV-negative	1	1

Treatment protocol				
Pediatric regimen (2RHEZ + 4RH)	1	1	0.172
Adult regimen (2RHEZ + 4RH)	1.61 (1.06–2.42)	0.023	0.73 (0.46–1.14)	

Year of TB initiation				
2018	1.61 (1.00–2.57)	0.048	1.25 (0.78–1.87)	0.311
2019	1.32 (0.80–2.15)	0.282	1.32 (0.80–2.15)	0.368
2020	1	1

**Table 5:** Factors associated with mortality among pediatric TB cases.

Demographic and clinical characteristics	Died *N* (%)	Alive *N* (%)	Univariate analysis		Multivariate analysis	
			COR (95% CI)	*p*-value	AOR (95% CI)	*p*-value
Total	73 (12.0)	512 (83.9)				

Gender						
Female	38 (52.1)	258 (50.4)	1.06 (0.65–1.74)	0.790		
Male	35 (47.9)	254 (49.6)	1		

Age group (years)						
0–4	33 (45.2)	212 (41.4)	1.17 (0.66–2.06)	0.576		
9-May	16 (21.9)	119 (23.2)	1.01 (0.52–1.99)	0.968		
10–14	24 (32.9)	181 (35.4)	1		

Residence						
Rural	16 (21.9)	128 (25.2)	0.83 (0.46–1.50)	0.544		
Urban	57 (78.1)	138 (74.8)	1		

Form of TB						
Extra pulmonary TB	21 (28.8)	148 (28.9)	1.48 (0.78–2.81)	0.230	0.49 (0.11–2.13)	0.379
PTB−	31 (42.5)	145 (28.3)	2.23 (1.23–4.03)	0.008	0.55 (0.13–2.37)	0.394
PTB+	21 (28.8)	219 (42.8)	1	1

Method of diagnosis						
Clinical diagnosis	53 (27.6)	294 (57.4)	1.96 (1.14–3.38)	0.015	2.22 (1.25–3.91)	0.006
Bacteriological diagnosis	20 (27.4)	218 (42.6)	1	1

Weight of child at TB initiation						
0–7 kg	16 (21.9)	65 (12.7)	1.59 (1.08–6.23)	0.033	2.24 (0.89–5.67)	0.087
8–14 kg	17 (23.3)	109 (21.3)	1.64 (0.70–3.86)	0.252	1.21 (0.49–3.00)	0.667
15–21 kg	12 (16.4)	110 (21.5)	1.15 (0.46–2.85)	0.760	0.91 (0.34–2.40)	0.859
22–28 kg	10 (13.7)	58 (11.3)	1.82 (0.69–4.74)	0.221	1.48 (0.53–4.07)	0.448
29–35 kg	09 (12.3)	75 (14.6)	1.26 (0.47–4.34)	0.633	1.29 (0.47–3.55)	0.619
>35 kg	9 (12.3)	95 (18.6)	1	1

HIV status						
HIV-positive	41 (56.2)	106 (20.7)	3.94 (2.29–6.76)	<0.001	4.23 (2.44–7.33)	<0.001
Unknown	06 (8.2)	141 (27.5)	0.43 (0.17–1.07)	0.072	0.44 (0.17–1.10)	0.081
HIV-negative	26 (35.6)	295 (51.8)	1	1

Treatment protocol						
Pediatric regimen (2RHEZ + 4RH)	48 (11.8)	358 (88.2)	1		
Adult regimen (2RHEZ + 4RH)	25 (14.0)	154 (86.0)	1.21 (0.72–2.03)	0.470		

Type of health facility						
Public	65 (89.0)	455 (88.9)	0.66 (0.18–2.38)	0.533		
Faith-based	5 (6.8)	43 (8.4)	0.54 (0.11–2.56)	0.440		
Private	5 (4.1)	14 (2.7)	1		

PTB+: bacteriologically confirmed pulmonary TB. PTB−: clinically diagnosed pulmonary TB.

## Data Availability

The data that support the findings of this study are available from the corresponding author upon reasonable request.
